# Socio-historical analysis of the social importance of pharmacovigilance

**DOI:** 10.3389/fsoc.2022.974090

**Published:** 2022-11-25

**Authors:** Juan R. Coca, Raquel Coca-Asensio, Gema Esteban Bueno

**Affiliations:** ^1^Unit for Social Research in Health and Rare Diseases, Faculty of Education of Soria, University of Valladolid, Soria, Spain; ^2^Department of Neurosciences (Pharmacology), University of Cadiz, Cadiz, Spain; ^3^Almeria Periphery Clinical Management Unit, Almería Health District, Andalusian Health Service, Almeria, Spain

**Keywords:** pharmacovigilances, sulfonamides, thalidomide, rofecoxib, biosocial

## Abstract

Pharmacovigilance is a scientific discipline that has changed a lot in recent years and is of great social importance. The case of the so-called sulfonamide elixir showed society the importance of this discipline. Since then, pharmacovigilance has evolved into a scientific discipline with a strong social character. In this paper, a historical review is made of several paradigmatic examples of this discipline to reflect on what pharmacovigilance could be like finally. We conclude that this discipline could be more closely related to other areas of the social sciences, which would help to promote a more democratic social environment taking into account the needs of individuals and social groups.

## Introduction

The development of pharmaceutical legislation began in the early 19th century in the United States, and by 1820 the *US Pharmacopeia* had published monographs that regulated compounding in the US (Haller, 1982). One of the earliest studies on the pharmacovigilance of the effects of chloroform was conducted by Gustave Darin (Caron et al., 2016). On the other hand, the first edition of the *British Pharmacopeia* was also published in the 19th century, specifically in 1864, in Europe. This pharmacopeia is the official collection of quality standards with which medicines in the UK must comply. It is produced by the *British Pharmacopeia Commission Secretariat* and depends on the British Medicines Agency (*Medicines and Healthcare Products Regulatory Agency*). The *British Pharmacopeia* incorporates monographs from the *European Pharmacopeia*, it is updated annually (the latest edition is dated 1st January 2015) and contains 3.000 monographs of substances and articles used in the practice of medicine. At the beginning of the 20^th^ century, in 1906, the *US Pharmacopeia* and the *National Formulary* acquired the status of legal pharmaceutical legislation in the United States (Kremers and Urdang, 1976). In the same year, the *US Food and Drugs Administration* (FDA), which was created in 1848, was established as a federal agency of the US government with the approval of the *Pure Food and Drugs Act* (Barkan, 1985), which was the first ratified law on drug regulation and consumer protection. This federal law prohibited the manufacture, sale or transportation of intoxicating medical products, among others, and it also required that certain substances such as alcohol, cocaine, heroin, morphine and cannabis would be appropriately labeled in terms of quality and quantity. The enforcement of this provision was only ensured in 1914, when the Harrison Narcotic Drugs Act was passed, prohibiting the sale of some narcotic drugs (Hansen and Dusenbury, 2007). Currently, the FDA is responsible for the regulation of food, drugs, medical devices (human and veterinary), cosmetics, biological products and blood derivatives. Its main function is to regulate medical products in a way that ensured the safety of US consumers and the effectiveness of marketed drugs (Weaver et al., 2008).

Pharmacovigilance (PV) is a disciplinary field that is often linked to epidemiological and pharmacological studies. This is because it is primarily seen as a discipline focused on assessments in the field of drugs approval and safety (Rocca et al., 2019). However, Rocca et al. (2019) are aware that this discipline has given rise to a number of new insights related to epistemology and epidemiology. Nevertheless, the generation of new strategies, methodologies and standards of evidence to enable the implementation of risk assessment is becoming increasingly relevant.

In this line, we wonder whether it is not somewhat limited to restrict pharmacovigilance to the aspects indicated by Rocca et al. (2019). They refer to social aspects, but they circumscribe them to the medical and scientific community. On the other hand, we consider that there are social phenomena (e.g., self-medication) that are related to the social understanding of reality and social perceptions. For this reason, we believe that PV is a broader concept and that it is closely related to social psychology, sociology and other disciplines. On this basis, we consider that historical studies allow us to analyse the processes that have been taking place in this field and, thus, to make decisions in this regard. Hence, the aim of this paper is to take a socio-historical look at some outstanding cases in order to understand the evolution of pharmacovigilance. We have focused our analysis on pharmacy professionals, being aware that the patient-partner is also an extremely important agent. However, in this research we believe that pharmacists play an important role in the social processes related to pharmacovigilance.

## The first pharmaceutical control systems: The case of sulfonamides

Domagk (1895–1964) demonstrated in 1932 the efficacy of sulfonamides for the treatment of streptococci. Subsequently, in 1935, the trademark patent Protonsil was established, allowing the subsequent marketing of the first drug with this active ingredient, which led to the production and marketing of the first sulfonamide (Morales and Bosch, 2007). The media dissemination of the efficacy of the first sulfonamides generated a great social impact. In fact, there was an imaginary element that conditioned the positive opinions of the drug. In 1936, the publication of news in the New York Times showing that President Roosevelt's son, after being admitted to hospital with a severe tonsil infection caused by *Streptococcus*, was cured by *Prontylin*, a dispensing form of Protonsil (Morales and Bosch, 2007). The commercial success of this drug led the *Food and Drug Administration* (FDA) to recognize the growing regulatory problem that was being generated by the expansion of sulfonamides (Cooper, 2002).

The success of sulfonamides led to widespread sales and the generation of commercial alternatives. One of the latter was the so-called “Sulfanilamide Elixir.” This preparation was developed in the use of 72% diethylene glycol (Wax, 1995). The issue was that the producing company (Massengill Corporation), as stated in a report of the Secretary of Agriculture of the United States of America published in 1937, did not test the toxicity of the ingredients and focused on evaluating the taste, color and labeling (Secretary of Agriculture, 1937). Furthermore, the company did not disseminate the presence of diethylene glycol in the product (Wax, 1995). This resulted in at least 107 deaths from ingestion of the product. Besides, the FDA was only able to blame Massengill with a trivial problem related to mislabeling of the product, since it was claimed to be an elixir when in fact it had an alcoholic content (Cooper, 2002). At the end of 1937, an editorial was published in (The British Medical Journal, 1937) reflecting on the sulfonamide problem. The text begins by stating: “A recent outbreak of poisoning in the USA has sensationally and tragically demonstrated the unexpected dangers which may arise in the introduction of the therapeutic use of chemical compounds without adequate preliminary testing of their possible toxic actions.” As a result of this problem and, because of the lack of regulations to control the production process, the United States congress enacted the United States Federal Food Drug and Cosmetic Act, a set of laws that granted the FDA authority to demand the safety of food, drugs and cosmetics. However, and despite the attempts to establish an analysis process and verification of pharmaceutical products, it was not until the end of the 20th century that well-established processes for defending society against the negative effects of certain drugs were in place. In fact, for Abraham (2008), the pharmaceutical sector escaped social scrutiny for many years, since in the later part of the 19th and 20th centuries, industrialized countries and society were seen as a kind of market for the products of an expanding scientific-medical industry. The fact that Massengill was only concerned with the commercial elements of his product is a proper evidence of this issue.

The United States Federal Food, Drug and Cosmetic Act is the beginning of a different perspective, as well as the intertwining of the social and the pharmaceutical. In fact, this regulation is the seed of the current pharmaceutical legislation focused on a preventive process that conditions the marketing of industrial products, and that requires tests on the safety of pharmaceutical products and also it grants the FDA surveillance powers after the products have been authorized for marketing (Silva-Ortiz, 2011). Nevertheless, and despite the implementation of a regulation that gave the US FDA more power, this did not prevent other similar events from occurring. As a matter of fact, on 19th March 1941, George Adams, the head of the Food and Drug Administration's Boston Station, found that three girls in his area went into a coma after taking fifteen grams of sulfathiazole (Swann, 1999). The problem was caused by a deficiency in manufacturing and in quality control related to the production of the drug. On 24th December 1940, analysts at the marketing company “Winthrop,” confirmed that some of their sulfathiazole, specifically the batch MP 29, was contaminated with Luminal, which was the brand of phenobarbital they produced (Swann, 1999). This issue arose because the company did not alert the FDA about the contamination, and thus, there was an inefficient recall of the affected batch. William Weiss, who was the chairman of the board of Sterling Products at that time, told the FDA that he thought Winthrop had not tried to conceal the contamination, but that it was possibly due to poor decision-making resulting from a misjudgement of the seriousness of the situation (Swann, 1999). Around 120.000 tablets of Winthrop's contaminated sulfathiazole were circulating in the United States with a subsequent risk to the population. Although the Sulfanilamide Elixir tragedy, which was the event that marked the beginning of the Food, Drug and Cosmetic Act of 1938, was still remembered by American society, Winthrop did not alert the FDA about the contamination.

It is possible to affirm that, in these early years of the development of the pharmaceutical industry, there were a series of events related to the lack of control processes and regulatory systems for production and marketing. These events were preceded by a positive image of the potential in the healing process of several pathologies (Morales and Bosch, 2007). This act could possibly have led to a certain over-optimism among the public regarding the benefits of chemical products, which on the other hand, they were not being controlled. Once the “Sulfanilamide Elixir” event occurred, social perception changed again, partly as a result of the information exposed in the various articles of the media at that time. It is conceivable to consider that, although the media played an important role, it cannot be forgotten other relevant element which explains the social behavior toward sulfanilamides: the economic crisis.

In 1929, a severe economic crisis emerged in the United States under the name of “The Great Depression,” whose effects had an impact on the life of American citizens and on their social perception. The socio-economic transformations of that time led to an increase in the number of suicides, although there was also a notable increase in the economy. On this account, President Roosevelt generated several measures aimed at greater state intervention in investment and the implementation of public works in order to relieve the effects of the crisis (Comín, 2012). During those years, the life expectancy at birth of US citizens varied substantially, showing very marked peaks. In 1936, specifically, a notable drop was shown in the life expectancy of women and men regardless of their origin (Tapia and Diez, 2009). Nevertheless, health indicators of the US population show that the collective health condition of the population improved at that time. However, for most older age groups, mortality tended to peak during the years of strong economic expansion (such as 1936–1937).

This social, economic and health reality has led to the current welfare state, in which the control of commercial products, that could have a negative impact on society, is of vital importance. Hence, it was at this historical moment that the seeds of the pharmaceutical controls that are known today were sown, but it was necessary to wait a few years for the germ of such systems to take full shape.

## The maturity of pharmaceutical control systems: The case of thalidomide

In 1954, the German company Chemie Grünenthal succeeded in obtaining the molecule alpha-phthalimido-glutarimide, known as thalidomide. This drug was classified as a sedative and hypnotic, and was used in 1957 for the treatment of anxiety, insomnia, nausea and vomiting in pregnant women (Martínez-Frías, 2012). In 1956, the first isolated case of phocomelia was documented after the exposure of thalidomide, and in the following 5 years, 3.000 cases of dysmelias, congenital malformations such as amelias, phocomelia or absence/hypoplasia of the thumb or fingers, among others, were gradually reported worldwide (Papaseit et al., 2013). However, in a short letter to the British Medical Journal, Florence (1960) indicates that patients treated with thalidomide for extended periods (8 months to 2 years) reported negative effects of thalidomide intake complaining of: (1) Paresthesia affecting first the feet and then the hands. (2) Coldness of the extremities and marked paleness of the toes. (3) Occasional slight ataxia. (4) Nocturnal cramps in the leg muscles. When the treatment was eliminated and the patients stopped taking the substance, the negative effects subsided. This led Leslie Florence to suspect the toxicity of thalidomide. Subsequently, in January 1962, The Lancet magazine published a series of letters of the effects of thalidomide. The first of these letters, which was signed by Lenz (1962), describing 52 children with malformations caused by the ingestion of this substance by their mothers during pregnancy. However, in this letter Lenz states that at a conference held on 18th November 1961, in which the author took part, they had already discussed the role of this substance in the development of human malformations. The same issue of The Lancet also published another letter by Pfeiffer and Kosenow (1962) in which he indicated the existence of a high statistical significance between the intake of thalidomide during the first trimester of pregnancy and the occurrence of defects. The third letter, which is signed by Hayman (1962), the managing director of the Distillers Company, begins by thanking them for the expressions of appreciation they received, and in which thalidomide is highlighted. He goes on to say that due to the small amount of data and official statistics, it is particularly difficult to establish the harmful effects of this substance. Irrespective of one's personal assessment of Hayman's writing, the objective data of the various researchers showed that thalidomide was not as harmless as it was claimed to be.

Papaseit et al. (2013) state that it was the Lenz letter that led to the withdrawal of thalidomide from the German market and its gradual elimination from the market worldwide (1961–1962). Salvador Coderch et al. (2014) state that this withdrawal was caused by an article published in the *Welt am Sonntag* newspaper on the 26th November 1961 discussing this issue. Grünental's action took place the following day, on the 27th November. It is difficult to establish a specific cause, since social reality is more complex than that and every social action is the result of a concatenation of events. Regardless of its origin, the process took time to reach Spain, which was one the last regions to officially ban its marketing as this took place in January 1963 (Papaseit et al., 2013). In Spain, a Ministerial Order was published on 18th May 1962 (Salvador Coderch et al., 2014) prohibiting the marketing of medicines containing thalidomide. Despite this, the Royal Decree 1006/2010 of the 5th of August states that there may have been some instances in the period between 1960 and 1965 in which “substances containing thalidomide could still be in circulation or in the possession of private individuals.”

In Spain, the social process generated by thalidomide has been particularly dramatic. This was caused by the denial of thalidomide sales by the Spanish authorities for more than 30 years. At that time this implied that there were supposedly no cases in Spain and, for this reason, it put those affected individuals in a situation of institutionalized helplessness, exclusion and marginalization. Currently, it has been estimated that there are between 1.500 and 3.000 new-borns with malformations (Papaseit et al., 2013). The seriousness derives from the lack of official registry, which has prevented affected individuals and families from accessing political and social recognition, as well as any financial compensation or health assistance.

The opposite pole to Spain is the United States. In that territory, no thalidomide patients were affected thanks to the caution of the FDA supervisor, Dr. Kelsey, who rejected the application for authorization to market such drugs. In view of conflicting information, the decision was made to wait for more data on its safety. For this reason, Dr. Kelsey was decorated by President Kennedy on 7th August 1962 with the “President's Award for Distinguished Federal Civilian Service” (Rajkumar, 2004). As a consequence of these events, on 10th October 1962, the United States Congress unanimously passed the Judiciary Committee's bill on amendments to the United States Federal Food, Drug and Cosmetic Act. In this amendment, an administrative procedure was established (Silva-Ortiz, 2011) for the authorization of clinical trials and the need to demonstrate the therapeutic efficacy of medicines before applying for marketing authorization (Silva-Ortiz, 2011). This regulation puts health before marketing, substantially institutionalizes the production of pharmaceuticals in the social context and strengthens what later became known as social medicine (Ryle, 1943, p. 635):

“In short, social medicine means what it says. It is to embody the idea of medicine applied to the service of man as *socius*, as companion or comrade, with a view to a better understanding and a more lasting help to all fundamental problems and contributing to the avoidance of active health, and not the mere removal or relief of a present pathology. Social medicine also embodies the idea of medicine applied to the service of the *societas*, or community of men, with a view to reducing the incidence of all preventable diseases and raising the general level of human physical fitness.”

The worldwide tragedy of thalidomide generated such a social effect that it led to a second sept toward the strengthening of voluntary adverse reaction reporting systems, which gave rise in 1963 to the International Pharmacovigilance Programme of the World Health Organization (WHO) with centers in 10 countries in that year. Since 1971, they have been under the authority of the world pharmacovigilance center (Caron et al., 2016). In Spain, the spontaneous adverse reaction reporting programme began in 1982 and, 2 years later, it joined the WHO programme.

## A third prominent example: Rofecoxib (Vioxx^®^

Rofecoxib is a non-steroidal anti-inflammatory drug that functions as a selective inhibitor of the enzyme cyclo-oxygenase-2 (COX-2) and thus of prostacyclin synthesis (Karha and Topol, 2004). Vioxx^®^ was a drug marketed by Merck Sharp & Dohme (MSD) and it was indicated for the symptomatic treatment of rheumatoid arthritis and osteoarthritis. In the United States of America, the FDA considered the benefit-risk assessment of the drug to be favorable and it granted marketing authorization on 20th May 1999 (Presley, 2009). In February 2001, the FDA prepared two reports on notifications of possible cardiovascular adverse events associated with Vioxx^®^. The FDA required only Merck to incorporate precautions in its labeling (Horton, 2004). The scientific community urged the FDA to request further clinical safety testing, but the FDA did not do so (Horton, 2004; Topol, 2004). The scientific community therefore considered that the FDA's actions were insufficient to prevent possible adverse drug reactions (ADRs). In this regard, studies and critical comments were published in various prestigious international scientific journals on the methodological deficiencies of the clinical studies carried out on Vioxx^®^, warning of its link to serious cardiovascular risks. The FDA only required Merck to incorporate a series of precautions in this respect in its labeling. Despite the doubts and deficiencies, on 20th July 2001, Merck Sharp & Dohme obtained marketing authorization for another drug with rofecoxib as an active ingredient, Ceoxx^®^, indicated for the symptomatic treatment of short-term acute pain and primary dysmenorrhoea. Publications warning about Vioxx^®^ ADRs were published from 2000 (Horton, 2004) to 2004. (Mukherjee et al., 2001; Horton, 2004; Jüni et al., 2004) In 2004, the serious ADRs associated with this drug became undisputedly evident. Merck notified the FDA of these findings and on 30th September 2004 voluntarily withdrew Vioxx^®^ and Ceoxx^®^100.

The unethical problem of the corporation is highlighted by a Wall Street Journal investigate journalism report revealing the existence of emails confirming the knowledge of the adverse cardiovascular effects of Vioxx^®^ by some Merck executives (Horton, 2004). On the other hand, harsh criticism of the FDA's performance led to calls for more power, control and independence for the FDA (The Lancet, 2005). In addition, Horton (2004) questioned the very structure of the institution, stating the too often the FDA considers the pharmaceutical industry to be its client and, therefore, a vital sources of funding for its activities. Then, this fact undermines the FDA's performance by failing to act as a sector of society in need of sound regulation.

## The social importance of pharmacovigilance

Pharmacovigilance (PV) in Spain, according to Royal Decree 577/2013 of 26th July, is defined as the public health activity whose objective is the identification, quantification, evaluation and prevention of risks associated with the use of medicinal products (RAM) once authorized. This implied that PV is a biomedical risk control activity and, potentially, it could be also a pharmacological social risk minimization activity. Additionally, it is an inherent part of the clinical use of medicines, and it starts during the pre-marketing phase of medicines, as well as it reaches its peak after their authorization and marketing. In fact, PV has been a discipline focused on the post-authorization and post-marketing period (Hartford et al., 2006). Nevertheless, this has gradually changed. PV, under the influence of biological disciplines, has evolved toward an anticipatory and proactive approach to the potential risks/benefits of medicines in the pre- and post- approval stages of drug development (Hartford et al., 2006).

Pharmacovigilance is of great relevance today. In fact, during the recent pandemic caused by the SARS-CoV-2 virus, it was crucial for the rapid commercialization of new drugs against this virus (Ellis and Toklu, 2020). Other outstanding examples of the importance of this discipline today are related to the use of opioids in the USA, to Levothyrox in New Zealand and France, or to the use of Ibuprofen in regions such as New Zealand or Spain. Likewise, the perspectives of analysis offered by the subsections of pharmacovigilance, such as cosmetovigilance and herbavigilance, are also remarkable (Toklu, 2016; Toklu et al., 2019). The examples are numerous and the challenges for this and other disciplines are proven to be enormous by negative consequences of ADRs.

ADRs are a major cause of morbidity and mortality, making the avoidance of ADRs extremely important for the population. In a classic study, Lazarou et al. (1998) analyzed 39 prospective studies conducted in US hospitals between 1966 and 1996. They found that ADRs accounted for 6.7% of hospital admissions and that they represented the sixth leading cause of death in the United States. On the other hand, currently the World Health Organization (Esteban et al., 2017) has established that adverse drug reactions (ADRs) are one of the 10 leading causes of death in the world.

Pharmacovigilance has undergone major changes since the thalidomide case. These changes were made mainly in the management of suspected cases of AMR (standardization of the processes for obtaining information, independence of sources, etc.), in the management of signals that raise suspicion for the detection of possible links between a given drug and its administration, and finally, in the management of the risk/benefit balance to implement processes that reduce risks for patients (Beninger, 2008).

Therefore, twenty-first century pharmacovigilance is not a discipline that simply discovers, reports and manages adverse events associated with approved and marketed drugs, but it is concerned with the systematic monitoring of the pre-marketing review process and post-marketing surveillance, which includes the use of drugs in everyday practice. However, all these considerations about pharmacovigilance are focused on the biomedical domain. Nonetheless, there are other actors involved in the systemic PV process that need to be taken into account and even explicitly incorporated into the PV process.

The first of these agents are pharmacists themselves. Obviously, as we have already indicated, in addition to pharmacists, physicians also have a preponderant role that should not be overlooked, especially general practitioners. Kumar (2017) notes that pharmacists' involvement in AMR reporting is, as he states, largely unknown. In fact, in the United States of America, a survey of 377 pharmacists in Texas found that 67.7% of the pharmacists surveyed had inadequate knowledge of the process of reporting to competent authorities (Gavaza et al., 2011).

The second major player that cannot be ignored in PV is society itself, which, through its interactions, could offer new opportunities for the management of PV-related information (Harpaz et al., 2014). This is because a large proportion of patients are often active participants in the exchange and dissemination of health-related information through social networks and, in particular, health social networks (Sarker et al., 2015). However, although the potential for obtaining useful information for PV is high, it is also necessary to bear in mind that the incorporation of data from social networks or everyday interactions between people generates serious drawbacks: credibility, timeliness, frequency, relevance, etc. (Sarker et al., 2015). On the other hand, the same author indicates that when trying to process natural language into computer language, it is found that consumers tend to use misspelled words, terms without medical correspondence and descriptive expressions to refer to health problems. Sarker et al. (2015) also indicate that a small proportion of drug-related data collected through social networks tends to contain information associated with AMR.

Therefore, pharmacovigilance also has an inescapable social component as it identifies previously unrecognized adverse events or changes in the patterns of these same effects, as well as the quality and adequacy of drug supply, and ensures effective communication with the public, healthcare professionals and patients about the risk/benefit balance and use of drugs (Pitts et al., 2016). Another important aspect of pharmacovigilance is centered around patient reports. These are often incomplete or unclear. In addition, there is also the possibility of reporting adverse drug effects *via* social networks (Paola and Claudio, 2020). Given this reality, we wonder if pharmacy professionals could play a more active role in regard to this by obtaining information directly from patients. Now, this aspect of pharmacovigilance has been traditionally done by relying on post-marketing spontaneous reporting systems (SRSs), such as: the EudraVigilance system (operated by the European Medicines Agency) or the Adverse Event Reporting System (US Food and Drug Administration, FDA). These systems gather voluntary reports produced by healthcare professionals, marketing authorization holders (MAHs) or consumers. However, the reporting rate of such systems is low, causing delays in the detection of ADRs (Pappa and Stergioulas, 2019). In this regard, several authors (Lardon et al., 2015; Bagheri et al., 2016; Sinha et al., 2018, among many others) have studied the usefulness of social media in pharmacovigilance. These works, together with other ones, show the enormous possibilities that exist in this social sphere. Sinha et al. (2018) even conclude that the FDA could develop strategies to more actively disseminate drug safety information through these social networks. They even argue that the FDA could benefit from information dumped on websites such as Wikipedia, which are frequently accessed for drug-related information. Most critical of such strategies, Lardon et al. (2015) suggest that there is a sufficient volume of pharmacovigilance data on social media to work with. However, they are aware that the quality of this information is variable and that further studies are needed to improve the process. For all these reasons, it could be concluded that these mechanisms are not yet sufficiently developed to be used with complete efficiency and reliability.

The use of social media to improve pharmacovigilance is one of the possible strategies of what has been called social pharmacology (Montastruc et al., 2021). This discipline, according to these authors, is the study of interactions between pharmaceuticals and society. In line with this Knight et al. (2017), a study on the pharmacovigilance of opioids, showed that social elements, mainly structural ones, affected opioid access. Similarly, in Canada, social groups have been found to be more prone to AMR. In fact, women have a lower proportion of ADRs (53.5%) compared to 60.9% of men. Furthermore, these authors also indicate that AMR have a direct social impact, i.e., they directly affect people's lives. For this reason, Castillon et al. (2019) suggest that social dimensions such as social and family functioning, psychological functioning, functioning related to daily life, and functioning at work or school should be included and assessed in AMR reporting. This gives an idea of the importance of the social aspects in pharmacovigilance. On the other hand, a study previously conducted (Pottie et al., 2008) found that the development of collaborative professional practices between pharmacists and physicians was beneficial because, among other things, it provided clinical safety for physicians. Well, we believe that, in a similar way, the collaboration of pharmacy professionals (but also of medicine) would allow us to broaden our understanding of the social world. One possibility would be to develop strategies for ongoing collaboration and communication when analyzing human behavior or social perceptions. Another more recent and enlightening study shows how it is possible to develop virtual forums (cyber-forums) to share information, motivate and understand the practical constraints that influence pharmacovigilance (Rochoy et al., in press). The question would then be similar: wouldn't it be desirable that sociologists could actively participate to help better understand the social and practical determinants of pharmacovigilance?

## Conclusion

For all these reasons, we believe that it is essential that the biomedical, pharmacological and social research fields interrelate in a more effective way. This would require a different strategy to the current one. In this sense, we could say that there would be several main actors involved: those who report ADRs, those who investigate ADRs, and those who study the mechanisms of prevention, education and social perception of medicines in order to understand ADRs and, if possible, reduce them. In addition, we believe that this interaction would allow the information obtained and issued by pharmacists (as well as other health professionals) to be previously filtered and have greater reliability than that coming, for example, from social networks.

On the other hand, the way in which the misperception of medicines, vaccines, etc., is generated in the citizenship would be better understood, and also the behaviors that promote or facilitate AMR could be more effectively reduced. We also assume that it would be easier to reduce the likelihood of self-medication or, at least, to increase the decision-making of those who choose to self-medicate.

Hence, and in this context, pharmacy would become an agent of socio-biomedical democratization, since it would act as a “translator” of citizens' impressions. However, in this hypothetical process of interrelation between pharmacists and society, it would also be of vital importance to establish mechanisms for dialogue with social scientists. The latter have a better understanding of social conditioning factors and could encourage better vigilance and greater social acceptance of pharmaceutical vigilance itself.

In any case, the challenges of PV in today's society are numerous and, in our opinion, it is clear that they require the incorporation of the greatest number of social agents that make possible a dynamic of constant information in order to implement flexible and appropriate control and management strategies. Finally, we believe that further research would be necessary to encourage the development and structuring of this process of interrelation that we are discussing.

## Author contributions

JC has written the definite article, and participated in the search for information and in the analysis. RC-A has written a first draft, and participated in the information search and analysis. GE has corrected the final article and has participated in the analysis of the information. All authors contributed to the article and approved the submitted version.

## Conflict of interest

The authors declare that the research was conducted in the absence of any commercial or financial relationships that could be construed as a potential conflict of interest.

## Publisher's note

All claims expressed in this article are solely those of the authors and do not necessarily represent those of their affiliated organizations, or those of the publisher, the editors and the reviewers. Any product that may be evaluated in this article, or claim that may be made by its manufacturer, is not guaranteed or endorsed by the publisher.
